# Chromosome Evolution in the Free-Living Flatworms: First Evidence of Intrachromosomal Rearrangements in Karyotype Evolution of *Macrostomum lignano* (Platyhelminthes, Macrostomida)

**DOI:** 10.3390/genes8110298

**Published:** 2017-10-30

**Authors:** Kira S. Zadesenets, Nikita I. Ershov, Eugene Berezikov, Nikolay B. Rubtsov

**Affiliations:** 1 The Federal Research Center Institute of Cytology and Genetics SB RAS, Lavrentiev ave., 10, Novosibirsk 630090, Russia; nikotinmail@mail.ru (N.I.E.); rubt@bionet.nsc.ru (N.B.R.); 2 European Research Institute for the Biology of Ageing, University of Groningen, University Medical Center Groningen, Antonius Deusinglaan 1, 9713AV, Groningen, The Netherlands; e.berezikov@umcg.nl; 3 Novosibirsk State University, Pirogova str., 2, Novosibirsk 630090, Russia

**Keywords:** whole genome duplication, karyotype evolution, intrachromosomal rearrangements, flatworms, *Macrostomum lignano*

## Abstract

The free-living flatworm *Macrostomum lignano* is a hidden tetraploid. Its genome was formed by a recent whole genome duplication followed by chromosome fusions. Its karyotype (2n = 8) consists of a pair of large chromosomes (MLI1), which contain regions of all other chromosomes, and three pairs of small metacentric chromosomes. Comparison of MLI1 with metacentrics was performed by painting with microdissected DNA probes and fluorescent in situ hybridization of unique DNA fragments. Regions of MLI1 homologous to small metacentrics appeared to be contiguous. Besides the loss of DNA repeat clusters (pericentromeric and telomeric repeats and the 5S rDNA cluster) from MLI1, the difference between small metacentrics MLI2 and MLI4 and regions homologous to them in MLI1 were revealed. Abnormal karyotypes found in the inbred DV1/10 subline were analyzed, and structurally rearranged chromosomes were described with the painting technique, suggesting the mechanism of their origin. The revealed chromosomal rearrangements generate additional diversity, opening the way toward massive loss of duplicated genes from a duplicated genome. Our findings suggest that the karyotype of *M. lignano* is in the early stage of genome diploidization after whole genome duplication, and further studies on *M. lignano* and closely related species can address many questions about karyotype evolution in animals.

## 1. Introduction

Recently, molecular cytogenetic studies performed on the free-living flatworm *Macrostomum lignano* revealed extended homologous chromosome regions, suggesting hidden tetraploidy, which arose as a result of the whole genome duplication (WGD) followed by chromosome rearrangements [1]. Comparison of karyotypes in closely related species indicated the relatively recent WGD that took place in the *M. lignano* evolutionary lineage. The largest chromosome (MLI1) was formed through the fusions of one chromosome set of the hypothetical ancestral karyotype, while others probably avoided drastic reorganization. As a result, the modern *M. lignano* karyotype consists of two parts containing similar DNA content, but differing in the number and morphology of their chromosomes. The only difference in DNA content discovered between MLI1 and the set of three pairs of small metacentrics was the loss of two clusters of pericentric and two clusters of telomeric repeats in the chromosome MLI1 [1]. Probably, some copies of these DNA repeats were conserved in MLI1, but a low number of conserved copies prevented their detection by fluorescent in situ hybridization performed with labeled telomeric DNA [2] and microdissected DNA probes [1]. Additionally, the high frequency of aneuploidy and worms with rearranged chromosomes were observed in the inbred lines of *M. lignano*. It was suggested that the *M. lignano* genome is in the early stages of genome diploidization after a WGD event [1]. We propose that a detailed analysis of the organization of individual chromosomes in *M. lignano* can allow us to elucidate the main trends and mechanisms of karyotypic and genome evolution taking place in the early stages of the evolution after WGD.

The data of the previous study hinted at the presence of extended continuous regions in the MLI1 chromosome homologous to all small metacentrics [1]. In this study, we focused on the comparative localization of unique DNA fragments and repeat clusters in metacentrics and in homologous regions of the chromosome MLI1. The data on chromosomal rearrangements taking place in the evolution of *M. lignano*, its closely related species *Macrostomum* sp. 8 obtained earlier [1,2] and described in the current study, and the mechanisms of their possible origin are discussed. The differences revealed in homologous regions are also considered as possible factors provoking chromosomes’ rearrangements, which led to the massive loss of duplicated chromosome regions after WGD.

We propose that *M. lignano* and its closely related species can be developed into a very useful and informative model for the investigation of the mechanisms of genome rediploidization after WGD. In contrast to the other species involved in similar studies of early genome evolution after WGD [3,4], these flatworms show small genome sizes and karyotypes consisting of a small number of chromosomes. The genome and transcriptome of *M. lignano* were recently sequenced [5,6,7]. All chromosome rearrangements and genome reorganizations that took place in the evolution of *M. lignano* can be easily identified and described in detail. We hope that the present work will help the further development in the studies of genome evolution in animals using these flatworms as model species.

## 2. Materials and Methods

### 2.1. The Samples of M. lignano

The free-living flatworms *M. lignano* were maintained and bred in the standard laboratory condition [8,9]. The karyotype of *M. lignano* (2n = 8) was previously described [10]. It consists of one pair of large and three pairs of small metacentric chromosomes. In this study, we used the specimens from the DV1/10 subline derived from the DV1 inbred line for studies on *M. lignano* chromosomes [1]. The inbred DV1 line was previously established via full-sibling and half-sibling inbreeding for 24 generations and has since been kept at small population sizes to maintain a high level of homozygosity [11]. The genome and transcriptome of worms from the DV1 line were sequenced and annotated [5,6,7,8,9]. Karyotyping of the DV1 line performed by chromosome analysis of more than 300 individual worms showed that the DV1 line contains euploid (2n = 8), as well as aneuploid (2n = 9 and 2n = 10) worms [2]. Aneuploids contained mostly one or two additional copies of chromosome MLI1. In this study, for chromosome preparation, the subline of the DV1 line was used. The DV1/10 subline of *M. lignano* mostly consists of aneuploid worms with the 2n = 10 karyotype (four large metacentrics and six small metacentrics) [1]. Here, we refer to the karyotype with four copies of the chromosome MLI1 (2n = 10) as the “normal” karyotype of the worms from the DV1/10 subline. Taking into account the high level of karyotype diversity in the DV1 line, we regularly karyotype the worms of the DV1/10 subline. The latest round of karyotyping of 150 randomly selected animals was carried out before the present study.

### 2.2. Chromosome Slide Preparation

For karyotyping, metaphase plates were prepared from individual worms with the standard technique [2]. For metaphase chromosome microdissection and for fluorescent in situ hybridization (FISH), chromosome preparation was performed with the cell suspension method [1]. For FISH experiments, the fixed cell suspension was dropped onto cold wet glass slides (76 mm × 26 mm × 1 mm). For metaphase chromosome microdissection, the fixed cell suspension was dropped onto clean cold wet cover slips (60 mm × 24 mm × 0.17 mm). Chromosome slides prepared for FISH were stored at room temperature and treated with 60 °C heating for two hours. Metaphase chromosome microdissection was carried out on the day of chromosome preparation after Giemsa staining.

### 2.3. Chromosome-Specific DNA Probes

The generation and characterization of microdissected DNA probes derived from MLI2 (Mli2) together with chromosomes MLI3 and MLI4 (Mli3_4) were described earlier [1].

### 2.4. Generation of Region-Specific Microdissected DNA Probes

**Figure 1:**
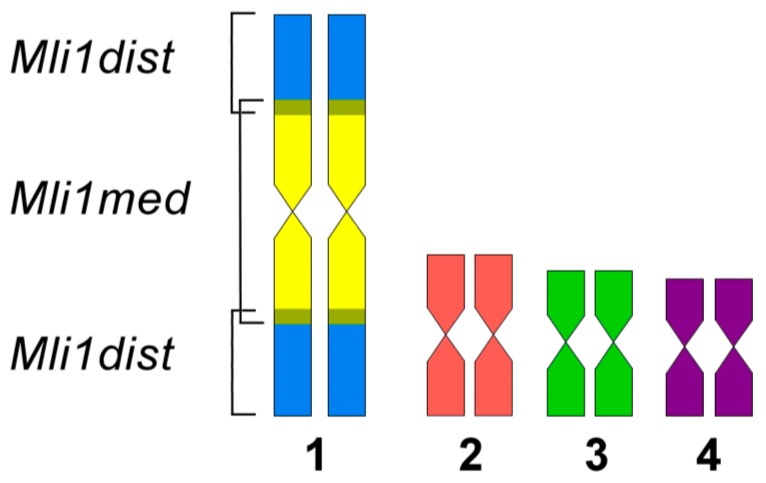
Scheme of the dissection of the MLI1 chromosome for generation of region-specific DNA probes. Medial part of MLI1 (in yellow color): Mli1med DNA probe; distal parts of MLI1 (in blue): Mli1dist DNA probe. Overlapping regions are shown in grey.

Metaphase chromosome microdissection and generation of DNA probes derived from distal and proximal regions of *M. lignano*’s largest chromosome were carried out as previously described [1]. In the chromosome MLI1, the ratio of the length of the q-arm to the length of the p-arm is equal to 1.15 ± 0.13 [2]. The arms cannot be reliably distinguished after Giemsa staining. For this reason, MLI1 was dissected for the generation of two DNA libraries. One of them, Mli1med, was derived from the proximal region of MLI1, while another, Mli1dist, was derived from distal regions of both arms of MLI1. For the microdissection, we used metaphase plates having only the “normal” 2n = 10 karyotype of the DV1/10 worms. The scheme of the MLI1 dissection is shown in Figure 1.

In total, 15 copies of both distal and medial parts of MLI1 were collected to generate DNA probes, Mli1dist and Mli1med, respectively. They were immediately processed through amplification by the GenomePlex Single Cell Whole Genome Amplification Kit (WGA4) (Sigma-Aldrich, St. Louis, MO, USA) and labeled in an additional 20 cycles of polymerase chain reaction (PCR) using the Whole Genome Amplification 3 (WGA3) kit (Sigma-Aldrich). Mli1med was labeled with Flu-12-dUTP, fluorescein-5(6)-carboxamidocaproyl-[5(3-aminoallyl)2′-deoxyuridine-5′-Triphosphate)] (Biosan, Novosibirsk, Russia), while Mli1dist was labeled with TAMRA-5-dUTP, 5-tetramethylrhodamine-dUTP (Biosan, Novosibirsk, Russia). Additionally, unlabeled PCR products were generated in additional PCR cycles and used for suppression of DNA repeats, as described earlier [1]. Finally, the probe mix contained 7.5 µL of each DNA probe labeled in additional PCR cycles (i.e., Mli1med, Mli1dist), 37.5 µL of each unlabeled DNA probe, and 3 µL of salmon sperm DNA (10 mg/ml) as a DNA carrier in hybridization mix.

### 2.5. Generation of Labeled Unique DNA Fragments for Fluorescent in situ Hybridization Detection of Homologous Sites in the Chromosome of M. lignano

For the selection of the unique DNA sequences for the application as molecular cytogenetic markers of homologous sites in chromosomes of *M. lignano*, contigs longer than 20 kb were selected from a draft 454-based *M. lignano* genome assembly [7] and overlapped with the PacBio-based ML2 draft genome assembly [5]. Regions consistently assembled in both genome drafts were further filtered to exclude repetitive regions, and for the selected regions longer than 20 kb, sets of primers were designed using Primer3 software [12]. Finally, primer sets were tested to obtain a PCR product of the correct size. The pairs of forward and reverse primers for five DNA fragments and the length of PCR products are shown in Table 1. DNA amplification was performed with long-distance PCR (LD-PCR) using the BioMaster LR HS-PCR-Color kit (Biolabmix, Novosibirsk, Russia). Five DNA probes were prepared by labeling of 1 µg of PCR products with the nick-translation kit (Invitrogen, Carlsbad, CA, USA).

### 2.6. Generation of 5S and 28S rDNA Probes

DNA probes for 5S and 28S rDNA were prepared by PCR. For amplification of the 1177-bp fragment from the 28S rDNA *M. lignano* gene, the WormA (5-GCGAATGGCTCATTAAATCAG-3) and WormB (5-CTTGTTACGACTTTTACTTCC-3) primers were applied as described [13]. The 1645-bp fragment from the 5S rDNA *M. lignano* gene was amplified in PCR with the 5SF (5-CACCGGTTCTCGTCTGATCAC-3) and 5SR (3-CAACGTGGTATGGCCGTTG-5) primers. The design of 5SF and 5SR primers was based on the draft of the sequenced *M. lignano* genome [5]. For the generation of 5S rDNA, 20 ng of genomic DNA were added to 20 µL of the PCR master mixture (Biolabmix) and amplified in 25 PCR cycles (30 s at 93 °C, 40 s at 58 °C, and 90 s at 72 °C) with initial denaturation of 2 min at 94 °C. The PCR products were labeled in an additional 25 cycles of PCR in the presence of Flu-dUTP or TAMRA-dUTP (Genetyx, Novosibirsk, Russia).

### 2.7. Fluorescence In Situ Hybridization

FISH of the 28S rDNA probe and labeled fragments of 5S and 28S rDNA on metaphase chromosomes of *M*. *lignano* was performed as described previously [1,2]. DAPI (4′,6-diamidino-2-phenylindole) staining was carried out according to the standard protocol [14].

### 2.8. Microscopic Analysis

Microscopic images of metaphase plates were captured and analyzed using a CCD camera installed on an Axioplan 2 compound microscope (Carl Zeiss, Oberkochen, Germany) equipped with filter cubes #49, #10 and #15 (Carl Zeiss) using ISIS v. 4 software (METASystems GmbH, Altlußheim, Germany) at the Inter-institutional Shared Center for Microscopic Analysis of Biological Objects (The Federal Research Center Institute of Cytology and Genetics the Siberian Brach of the Russian Academy of Sciences, Novosibirsk, Russia).

## 3. Results

### 3.1. Characterization of the DV1/10 Subline before the Microdissected DNA Probes’ Generation and Fluorescent in situ Hybridization Experiments

**Figure 2:**
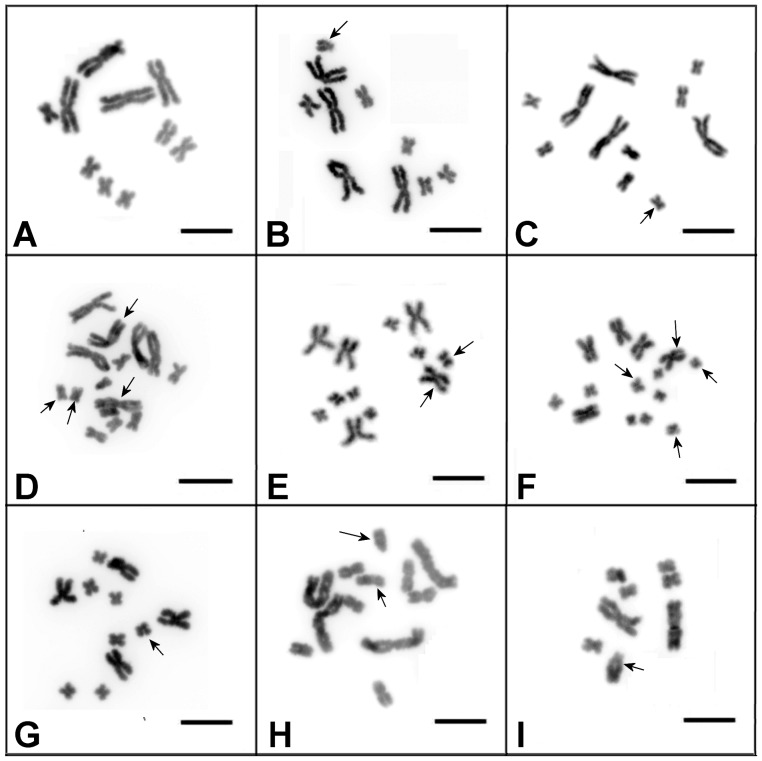
Metaphase plates from the worms of the DV1/10 inbred subline with the “normal” karyotype (2n = 10; four large and six small metacentrics) (**A**) and with abnormal karyotypes (**B**–**I**). (**B**) 2n = 10; four large, five small metacentrics, one small acrocentric chromosome (indicated by the arrow); (**C**) 2n = 11; four large and seven small metacentrics (additional small chromosomes indicated by the arrow); (**D**) 2n = 15; four large and nine small metacentrics (additional chromosomes indicated by the arrow); (**E**) 2n = 12; five large and seven small metacentrics (additional chromosomes indicated by the arrow); (**F**) 2n = 14; five large and nine small metacentrics (additional chromosomes indicated by the arrow); (**G**) 2n = 11; four large and seven small metacentrics (additional metacentric indicated by the arrow); (**H**) 2n = 12; four large and seven small metacentrics and one small acrocentric chromosome (additional metacentric and acrocentric indicated by the arrow); (**I**) “abnormal” 2n = 8; two large metacentrics, one medium-sized acrocentric chromosome (acrocentric indicated by the arrow) and five small metacentrics. Chromosomes were stained with DAPI (4′,6-diamidino-2-phenylindole) (inverted images are shown). Scale bar: 10 µm.

Among 150 karyotyped worms, 142 had the expected 2n = 10 karyotype (Figure 2A). Of the other eight, five worms had numerical chromosomal abnormalities involving different chromosomes (Figure 2C–G), while the remaining three worms had structurally rearranged chromosomes (Figure 2B,H,I). The previously performed karyotyping of 200 worms from the DV1/10 subline also revealed abnormal karyotypes in eight worms [1].

All abnormal karyotypes were characterized by different aneuploidies and structural chromosomal abnormalities. The worms characterized with the “normal” karyotype for the DV1/10 subline and with observed abnormal karyotypes showed phenotype and behavior identical to those of worms with the standard *M. lignano* karyotype (2n = 8). No morphological or behavioral abnormalities were revealed in these worms.

### 3.2. Generation of Region-Specific Microdissected DNA Probes and Chromosome Painting

**Figure 3:**
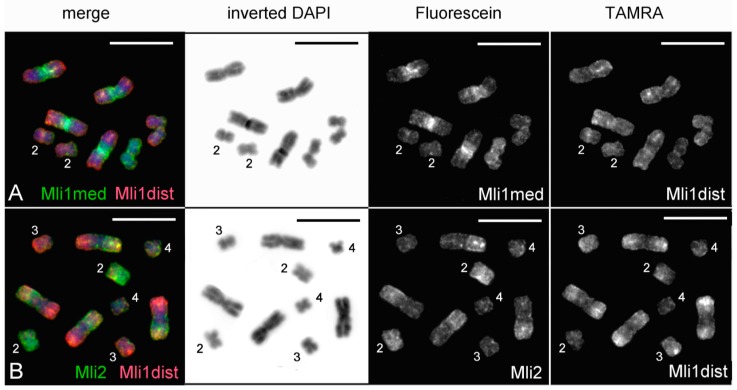
Painting and fluorescent in situ hybridization (FISH) on chromosomes of metaphase plates. (**A**) Mli1med (green) and Mli1dist (red) DNA probes; (**B**) Mli2 (green) and Mli1dist (red) DNA probes. Metaphase chromosomes were counterstained with DAPI (blue). Scale bar: 10 µm.

Chromosome MLI1 was easy to identify on metaphase plates after Giemsa staining, since it is substantially larger than the other chromosomes. According to the results of FISH performed using microdissected DNA probes generated from chromosomes MLI1, MLI2, and MLI3_4 and the labeled 28S rDNA, all four copies of the largest chromosome in worms of the DV1/10 subline were identical to MLI1 in the standard *M. lignano* karyotype [1]. The obtained DNA probes, Mli1med derived from the medial part of MLI1 and Mli1dist derived from distal regions of both arms (MLI1p and MLI1q), painted medial and distal regions, respectively, with overlaps in the border regions (Figure 3A). Additionally, the Mli1med probe intensively painted whole chromosome MLI4 and the distal region in one arm of both MLI2 and MLI3 chromosomes. Besides the distal regions of both arms of MLI1, the Mli1dist probe also painted the whole one arm and partly another arm of MLI3. In MLI2, Mli1dist also painted the whole one arm and partly another arm (Figure 3A). Two-color FISH carried out with different combinations of microdissected DNA probes (Mli1med and Mli1dist; Mli2 and Mli1dist) provided the unique painting patterns for small metacentrics (MLI2-MLI4), as well as for the arms of MLI1 (Figure 3A,B).

**Figure 4:**
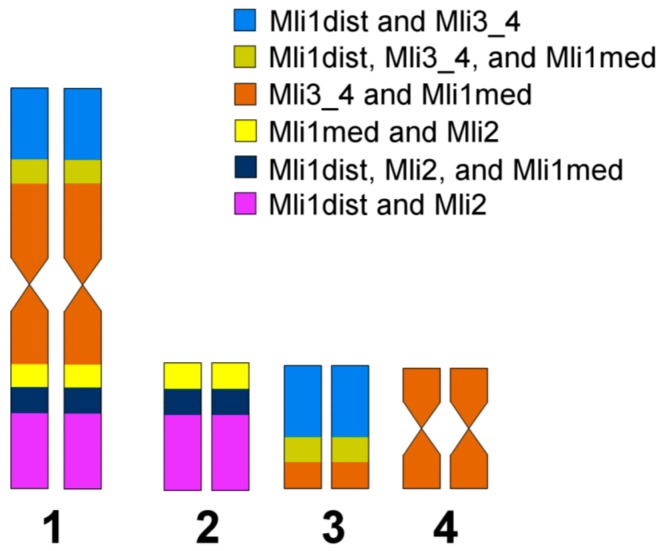
Scheme of chromosomal regions in the *M. lignano* chromosomes based on painting patterns of microdissected DNA probes. The regions marked with different colors were painted with different combinations of DNA probes. They were drawn approximately according to the estimation of their size. Microdissected DNA probes derived from the chromosome region provided FISH signal smooth fading in the region borderline. As a result, the position of centromeres in chromosomes MLI2 and MLI3 was not determined precisely in colored bands. For this reason, the centromeres of chromosomes MLI2 and MLI3 were not shown on the scheme.

**Figure 5:**
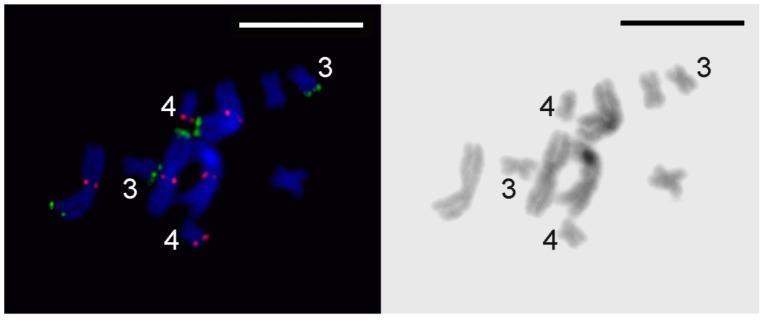
Localization of the 28S rDNA cluster (green) and the unique DNA fragment R4 (red) in chromosomes of *M. lignano*. The image with inverted DAPI staining is to the right of the FISH image. Metaphase chromosomes were stained with DAPI (blue). Scale bar: 10 µm.

We should note that short and long arms of the *M. lignano* chromosomes cannot be identified by a comparison of their lengths. Therefore, we named and identified them according to the painting patterns obtained with the sets of DNA probes. The MLI1p was painted with the Mli1med in the medial region, with Mli1dist in the distal region, and both of these DNA probes painted the region on the border of medial and distal regions. Additionally, the MLI1p was painted with Mli3_4 (Figure 4). The MLI1q showed the same painting pattern provided with the Mli1med and Mli1dist, but the large distal region of MLI1q was painted with Mli2, while its remaining proximal part was painted with Mli3_4. The whole chromosome MLI2 was painted with the Mli2 probe, and its whole q-arm and part of the p-arm were painted with the Mli1dist, while the remaining region of the p-arm was painted with the Mli1med. The whole chromosome MLI3 was painted with the Mli3_4 probe. Its p-arm and part of the q-arm were additionally painted with the Mli1dist, while the remaining part of the q-arm was painted with the Mli1med [1]. The painting patterns and their scheme are shown in Figure 3 and Figure 4. Painting with microdissected DNA did not allow us to distinguish MLI4p and MLI4q. This task was instead performed with FISH of the R4 probe containing unique DNA sequences (Figure 5). FISH with the R4 DNA probe marked an arm of the chromosome MLI4. We denoted this arm as MLI4q.

According to the FISH patterns, the regions of MLI1 homologous to small metacentrics appeared to be contiguous, but the question about existing inversions inside these regions and/or between them remains open. The implicit data in favor of rearrangements that differentiate small metacentrics from homologous regions of MLI1 were obtained from the analysis of worms with abnormal karyotypes.

**Figure 6:**
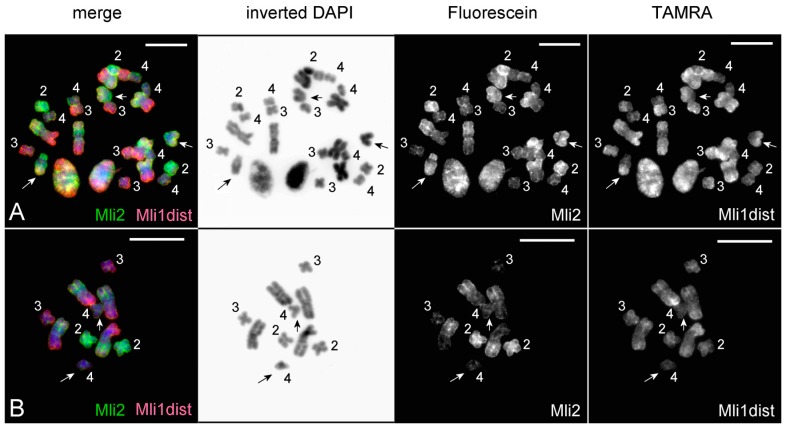
FISH using Mli2 (Fluorescein, green) and Mli1dist (TAMRA, tetramethylrhodamine, red) on abnormal metaphase plates of *M. lignano*. (**A**) Three metaphase plates with the abnormal 2n = 8 chromosome set (two large metacentrics, one unpaired medium-sized acrocentric, and five small metacentrics); (**B**) abnormal 2n = 10 chromosome set (four large metacentrics, four small metacentrics, and two small acrocentrics). Rearranged chromosomes are marked with arrows. Scale bar: 10 µm.

Painting with microdissected DNA probes was applied for the analysis of rearranged chromosomes, which were revealed in the karyotyped worms. In one of the animals, the karyotype consisted of eight chromosomes: two large metacentrics, five small metacentrics, and one unpaired medium-sized acrocentric (Figure 2I and Figure 6A). Painting of these abnormal metaphase plates with the Mli2 and Mli1dist probes revealed two copies of chromosomes MLI1, MLI3, and MLI4, one copy of MLI2, and a rearranged chromosome consisting of MLI1q and the distal part of MLI2p (Figure 6A). Painting with the Mli2 and Mli1dist probes identified the q-arm of acrocentric as the MLI1q, while the p-arm of the rearranged chromosome was painted only with the Mli2 probe. The mechanism for the rearrangements resulting in the formation of this acrocentric chromosome could be based on intrachromosomal rearrangements that differentiate chromosomes MLI2 and MLI4 from homologous regions in MLI1. To reveal these differences, FISH with unique DNA fragments from the *M. lignano* genome was performed, as described below. In another worm with the abnormal karyotype, the chromosome number was 10 (four large and four small metacentrics and two small acrocentrics) (Figure 6B). Painting using the same set of DNA probes showed that both acrocentrics consisted mostly of regions homologous to MLI4.

### 3.3. Comparative Analysis of Small Metacentrics and Homologous Regions in MLI1 with Fluorescent In Situ Hybridization of Unique Labeled DNA Fragments and rDNA Repeats

**Figure 7:**
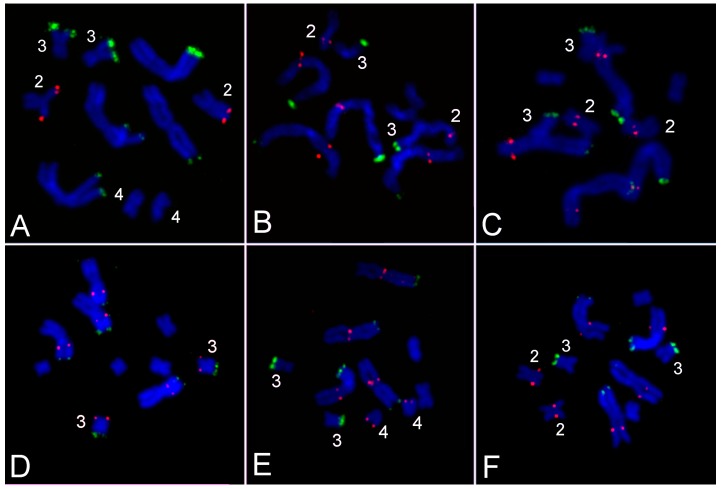
Localization of rDNA repeats and unique DNA fragments. (**A**) Clusters of 28S (green) and 5S (red) rDNA; (**B**) 28S rDNA (green) and unique DNA sequence R1 (red); (**C**) 28S rDNA (green) and unique DNA sequence R2 (red); (**D**) 28S rDNA (green) and unique DNA sequence R3 (red); (**E**) 28S rDNA (green) and unique DNA sequence R4 (red); (**F**) 28S rDNA (green) and unique DNA sequence R5 (red) in the *M. lignano* chromosomes. Metaphase chromosomes were stained with DAPI (blue).

**Figure 8:**
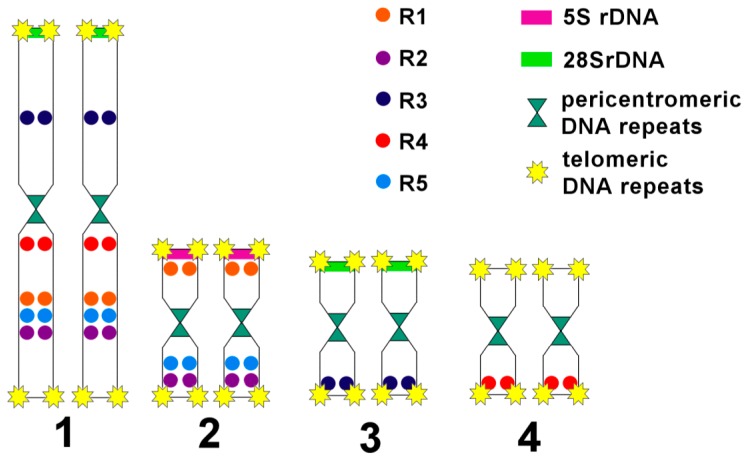
Scheme of the localization of the physical markers in the *M. lignano* chromosomes based on five unique DNA fragments and repeat clusters (5S and 28S rDNA, telomere and pericentromeric DNA repeats).

For detection of chromosomal rearrangements that could reshuffle DNA in the revealed homologous regions in the *M. lignano* chromosomes, FISH using labeled unique DNA fragments and fragments of the 5S and 28S rDNA was performed (Figure 7 and Figure 8). Previously, the 28S rDNA clusters were localized at the end of the arm of both the MLI1 and MLI3 chromosomes [2]. FISH with the labeled fragment of 28S rDNA carried out in this study confirmed our previous findings. According to the suggested nomenclature of chromosome arms that is based on the DNA probe painting, the 28S rDNA cluster was located at the end of the MLI1p and MLI3p. The cluster of 5S rDNA was localized at the end of the MLI2p. In contrast to 28S rDNA [2], the 5S rDNA cluster did not vary in size in the chromosomes of analyzed worms. We should note that the 5S rDNA cluster was not revealed in MLI1q despite its extended region showing high homology to MLI2. We cannot exclude that several copies of 5S rDNA repeats can be present in the MLI1q, as well as in other locations, but they were not detected by the FISH technique used in this study.

Fluorescent in situ hybridization with five labeled unique DNA fragments marked the homologous sites in MLI1 and other chromosomes (Figure 5 and Figure 7). Each of them was located at two sites; one was in one of the small metacentrics, and another one was in MLI1. Moreover, all of them were found in the regions of MLI1 homologous to small chromosomes containing corresponding DNA fragments. In all cases, on metaphase plates, we observed FISH signals on all four MLI1 copies and one pair of small metacentrics. Using FISH with different DNA probes, we identified chromosome arms of MLI1, MLI2, and MLI3 according to the painting patterns of the used DNA probes. In MLI4, chromosome painting using these DNA probes did not allow us to distinguish MLI4p and MLI4q. We named the arm with the unique DNA fragment R4 as the q-arm. The summarized results of physical mapping of the unique DNA fragments and repeat clusters are shown in Figure 7. Localization of the unique DNA fragments inside small metacentrics and in the homologous regions in MLI1 appeared to be a little different (Figure 7). For instance, the DNA fragment R4 was located at the end of MLI4q, while the second site of its location was found near the centromere of MLI1, suggesting that inversion containing the R4 DNA fragment took place during karyotype evolution in MLI4 or its homologous region in MLI1. Three unique fragments, R1, R2, and R5, were mapped into MLI2. The fragment R1 was localized in MLI2p, while signals from both R2 and R5 were found far away in MLI2q. In MLI1q, they were located close to each other.

Taking into account the loss of two clusters of DNA repeats homologous to the pericentromeric DNA, two clusters of telomeric repeats [1] and the cluster of 5S rDNA genes in the chromosome MLI1, we concluded that MLI1 differs from the sum of small metacentrics with deletions of five regions and with two intrachromosomal/intraregional rearrangements.

## 4. Discussion

Whole genome duplication is an evolutionary event that played an important role in the diversification of most eukaryotic lineages [15,16,17]. The process of rediploidization of duplicated genomes taking place after WGD is a key stage in the evolution of many phylogenetic lineages. The study of this stage requires comparative genomic analysis in species belonging to taxa containing species that recently underwent WGD (post-WGD species) and species that did not undergo WGD (pre-WGD species). The genus *Macrostomum* is one of these taxa. The chromosome number of these flatworms varies from three pairs to six pairs of chromosomes that are similar in size. In contrast to karyotypes of most *Macrostomum* species, the *M. lignano* karyotype consists of four chromosome pairs including the largest chromosome containing the extended regions homologous to intact small chromosomes. These data allow us to suggest that a recent WGD event took place in the *M. lignano* genome evolution and was followed by chromosome fusions. The small difference between the DNA content of MLI1 revealed by FISH and the sum of other chromosomes supports the suggestion that the *M. lignano* genome is at the early evolutionary stage towards its rediploidization, which is usually accompanied by chromosomal rearrangements and massive loss of duplicated genes.

In general, there are two mechanisms of the WGD, auto- and allo-polyploidization [18,19]. Autopolyploids arise through the species genome duplication of the same species, i.e., the newly formed duplicated genome contains four copies of each chromosome. In contrast, allotetraploids arise through genome duplication of interspecies hybrids and, therefore, contain two sets of divergent parental genomes [19]. At the first stage, neopolyploids as the usual tetraploids face the burdened of the typical meiotic problems (incorrect conjugation of chromosomes, multivalent formation, etc.) [18]. However, in allopolyploids, there are two complementary systems for further rediploidization. Firstly, the differentiation of homologous chromosomes (structural changes, DNA sequence divergence) leads to the preferential pairing of chromosomes derived from the same parent [20]. Secondly, a genetic control can distinguish between the differentiated sets of chromosomes and preclude pairing between homologous chromosomes (for instance, *ph1* in wheat) [18,21]. As a result, the meiotic problems might partially be solved through these systems. Can the genomic instability in inbred lines of *M. lignano* be considered as an indication of autopolyploidization in the genome evolution of *M. lignano*? We should note that genetic control could be reduced or disturbed by gene mutations and their homogenization as a result of inbreeding. Thus, the stage of increased genomic instability can appear also in the evolution of allotetraploids, and karyotypic instability in inbred lab-reared lines of *M. lignano* cannot be an argument in favor of the hypothesis of WGD taking place in the *M. lignano* phylogenetic lineage through autotetraploidy.

In neopolyploids, meiosis errors cause the formation of aneuploid gametes [18,22]. Therefore, it is not surprising that the frequently observed karyotypic abnormality in lab-reared lines of *M. lignano* was aneuploidy. At least three possible reasons for the high frequency of aneuploidy on MLI1 can be considered: (a) in meiosis, MLI1 containing paralogous regions is more often involved in abnormal chromosome conjugation than small chromosomes, followed by incorrect chromosome segregation and the formation of aneuploid daughter cells; (b) additional copies of MLI1 do not disturb the delicate gene balance, and these aneuploids are not eliminated by negative natural selection; (c) aneuploidy on MLI1 could confer a selective advantage under certain environmental conditions. It is possible that these reasons together can take place in natural populations.

Besides chromosomal rearrangements, the high frequency of additional structural chromosomal rearrangements was revealed in some inbred lines of the *M. lignano* [2]. Chromosomal instability in these lines could be induced by the decreased level of genetic diversity, which could lead to homozygous mutations in genes responsible for the correct meiotic process. As a result, the increased frequency of missegregation of chromosomes containing the extended homologous chromosomal regions can be observed. Indeed, worms in some inbred lines showed a stable normal karyotype, while in others, they were characterized by surprisingly high karyotypic diversity and newly formed chromosomal abnormalities [2].

In many cases of polyploidy, genome instability persists until the genome returns to the functionally normal ploidy level through gene loss, mutations, and chromosomal rearrangements, providing the difference between copies of duplicated chromosomes and preventing meiosis errors [22,23,24,25,26,27,28,29]. According to data on karyotype instability in lab-reared lines of *M. lignano*, the stage of genome instability after WGD can include the condition when the karyotype looks to be diploid, but actually contains the extended chromosomal regions with divergent paralogous genes. At this stage of evolution, conjugation followed by recombination of already rearranged chromosomes could lead to additional chromosomal rearrangements. In *M. lignano*, we observed similar rearranged chromosomes that probably were formed as a result of conjugation of MLI2 or MLI4 with homologous regions in MLI1 followed by recombination inside paralogous regions (Figure 6).

To date, the comparison of small metacentrics (MLI2-MLI4) with MLI1 revealed at least partial deletions of pericentromeric, telomeric and 5S rDNA repeats, as well as some intrachromosomal rearrangements differentiating small metacentrics from paralogons in MLI1. We did not reveal the extensive chromosomal reshuffling of paralogous regions of the *M. lignano* karyotype. The chromosomal rearrangements could take place in the largest chromosome, as well as in small metacentrics. We should note that chromosome evolutionary breakpoint regions (EBRs) are usually characterized by enrichment of repetitive elements, structural variants and/or segmental duplications [30,31]. Despite the revealed deletion of some clusters of DNA repeats (as was mentioned above) in MLI1, they could be involved in chromosomal rearrangements, restricting them to the regions homologous to small chromosomes. However, we should remind the reader that the difference between MLI2 and the homologous region in MLI1 was a result of rearrangement(s) involving either MLI2 or the correspondent region in MLI1.

The comparative cytogenetics of other species closely related to *M. lignano* that underwent WGD can probably reveal species at different stages of genome diploidization, characterized by different levels of duplicate-gene loss. For this comparative analysis, a more detailed study of the *M. lignano* genome is very important. The search for duplicated genes can be performed by sequencing of DNA libraries generated from individual chromosomes. Identification of such genes will allow the estimation of patterns of gene expression for paralogous genes. However, the loss of duplicated genes in the *M. lignano* genome cannot be very extensive. The study of worms having karyotypes with structurally rearranged chromosomes is probably a more promising approach. More detailed investigation of the morphology of these worms will possibly allow discovering abnormalities associated with chromosome rearrangements and provide more information on the mechanisms of the substantial elimination of duplicated gene copies observed in many phylogenetic lineages after WGD.

We should mention that genome reorganization in flatworms was recently revealed by another approach. An analyses of transcriptome data for 29 flatworm lineages showed that the effect of gene losses and gains taking place in genome evolution of flatworms could be mitigated by numerous hidden orthologs [32]. Additionally, it was shown that 14–30% of the putative hidden orthologs (among 3427 orthologous genes) identified in Tricladida are fast evolving paralogs [32]. Altogether, the findings obtained after genome and transcriptome analyses of free-living and parasitic flatworms indicated rapid genome evolution in flatworms [32,33]. It was also possible that transposable elements, which are common in flatworms [34,35,36], could generate additional genetic diversity, providing hidden orthology. The studies of the species-specific hidden orthologs revealed that the heterogeneity of their gene complements could be provided by different rates of ortholog evolution [32,37]. The study of the *M. lignano* genome reorganization as a whole and rearrangements of its individual chromosomes can provide valuable information for better understanding of the important points in the evolution of animal genomes.

## Figures and Tables

**Table 1:** Primers used for the amplification of unique DNA sequences.

Unique DNA Fragment		Primer Sequence	PCR Product Length
R1	F	CGGTACTCCTTCCAGGACATTG	20,124 bp
	R	CGCAACAGTGACGCTAACTATC	
R2	F	CACGTAGACGGTCATACGAGTT	20,232 bp
	R	CCGCTAATCCACGTCATGACTA	
R3	F	CTGACTGGGTGCGAATCCAT	20,254 bp
	R	AAACCTATGTCCTAGCTGTGCG	
R4	F	GTCCTGGAACTGTCTGTACACC	20,200 bp
	R	GTTTCGGGCAAACGATAGCTAC	
R5	F	AAAAATCTGCCGCCATTTGTGA	20,278 bp
	R	ATGAATGTTGACTCGGAAGGCT	

All primers are 5′-3′. F: forward; R: reverse.
